# Comparing Self-Reported Running Distance and Pace With a Commercial Fitness Watch Data: Reliability Study

**DOI:** 10.2196/39211

**Published:** 2024-01-04

**Authors:** Garrett Bullock, Joanne Stocks, Benjamin Feakins, Zahra Alizadeh, Amelia Arundale, Stefan Kluzek

**Affiliations:** 1 Wake Forest School of Medicine Winston-Salem, NC United States; 2 University of Nottingham Nottingham United Kingdom; 3 University of Oxford Oxford United Kingdom; 4 Department of Sports Science and Exercise Medicine Imam Khomeini Hospital Complex Tehran University of Medical Sciences Tehran Iran; 5 Red Bull Athlete Performance Center Thalgua Austria

**Keywords:** GPS, Garmin, training load, running, exercise, fitness, wearables, running, running distance, pace, pace distance

## Abstract

**Background:**

There is substantial evidence exploring the reliability of running distance self-reporting and GPS wearable technology, but there are currently no studies investigating the reliability of participant self-reporting in comparison to GPS wearable technology. There is also a critical sports science and medical research gap due to a paucity of reliability studies assessing self-reported running pace.

**Objective:**

The purpose of this study was to assess the reliability of weekly self-reported running distance and pace compared to a commercial GPS fitness watch, stratified by sex and age. These data will give clinicians and sports researchers insights into the reliability of runners’ self-reported pace, which may improve training designs and rehabilitation prescriptions.

**Methods:**

A prospective study of recreational runners was performed. Weekly running distance and average running pace were captured through self-report and a fitness watch. Baseline characteristics collected included age and sex. Intraclass correlational coefficients were calculated for weekly running distance and running pace for self-report and watch data. Bland-Altman plots assessed any systemic measurement error. Analyses were then stratified by sex and age.

**Results:**

Younger runners reported improved weekly distance reliability (median 0.93, IQR 0.92-0.94). All ages demonstrated similar running pace reliability. Results exhibited no discernable systematic bias.

**Conclusions:**

Weekly self-report demonstrated good reliability for running distance and moderate reliability for running pace in comparison to the watch data. Similar reliability was observed for male and female participants. Younger runners demonstrated improved running distance reliability, but all age groups exhibited similar pace reliability. Running pace potentially should be monitored through technological means to increase precision.

## Introduction

Physical activity is an essential component of a healthy lifestyle [1]. There is a substantial body of evidence highlighting the physical, social, and psychological health benefits of regular physical activity [1-3]. Sustainable physical activity interventions are needed, given that 31% of the global population is sedentary [4]. The World Health Organization’s physical activity action plan [5] identifies sport as an underused yet significant contributor to physical activity.

One widely popular sport globally is running [6]. Over the past 40 years, running has become one of the most popular physical leisure activities [7,8]. An estimated 50 million people in Europe participate in running as a way to stay healthy [9]. Due to high running participation prevalence [9], researchers have attempted to quantify running habits and training load, most notably through self-report [10]. Running load or workload is the distance run in 1 session. A training session is 1 running bout. Running speed is the intensity at which one runs for 1 running session [9,10]. However, there are potential inaccuracies from over–self-reporting due to recall bias [11] and social desirability of higher levels of physical activity [12], with potential differences by sex and age groups [13]. Further, the reliability of self-reported running pace has not been investigated, which is an important factor in quantifying running training intensity [14]. Due to these issues, research has investigated the reliability of wearables in quantifying running load [15]. Wearables, such as accelerometers, have demonstrated excellent reliability in assessing gait patterns, acceleration, and velocity [15].

Although wearable accelerometers are ubiquitously used in the general population [16] and are reliably used in research to measure physical activity levels [17], they are rarely used by running populations to track running load and training [18]. Runners opt for wearable GPS watches to track running training [19], with up to 90% of regular runners using some form of GPS monitoring when running [18]. GPS wearable technology quantifies running workload and speed [20]. A systematic review determined that there is excellent reliability for step counting and moderate validity for energy expenditure and distance run [21]. The most popular GPS wearable technology used by runners is the Garmin watch, as indicated by a previous survey where 44% of GPS and sports watches were Garmin, compared to 27% for Polar and 7% for Nike watches [22].

There has been previous related work in evaluating the reliability of running self-reports in large samples. In a sample of 92 endurance runners, followed for a 52-week (ie, 1 year) period, 93% of the runners participated in the entire follow-up period [10]. In a study of 53 running participants over 18 weeks, the response rate was 73% over the reporting period [23]. Another study surveyed 228 runners for at least 6 months, with a 2.2% attrition rate [24].

Although there is substantial evidence investigating the reliability of running distance self-reporting [10] and GPS wearable technology [20], there are currently no studies investigating the reliability of participant self-reporting in comparison to GPS wearable technology in running populations. There is a critical sports science and medical research gap due to a paucity of reliability studies assessing self-report running pace. Further, as GPS wearable technology is expensive [25], there may be a barrier for some recreational runners, decreasing the efficacy of using GPS monitoring alone to assess running workload [22]. Therefore, this study aimed to assess the reliability of weekly self-reported running distance and pace compared to a commercial GPS fitness watch, stratified by sex and age. These data will give clinicians and sports researchers insights into the reliability of runners’ self-reported pace, which may improve training designs and rehabilitation prescriptions.

## Methods

### Study Design

**Figure 1:**
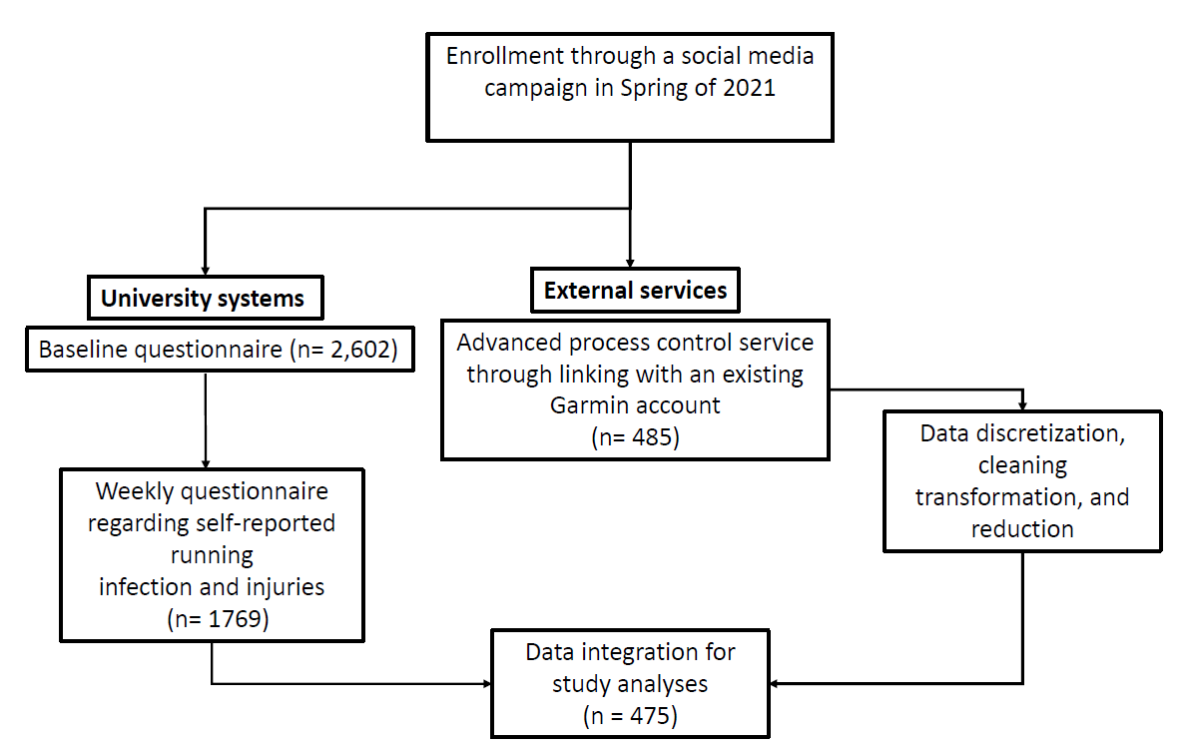
Participant Flow Diagram.

A prospective cohort study was conducted using a mobile-based app. Participants accessed a dynamic digital consent form through the app or the recruitment website during the spring of 2021 over a 4-month period. During consent, participants could select different levels of study engagement. All levels of engagement involved the following: (1) an acknowledgement, understanding, and consent to participate in the study; (2) a baseline questionnaire collecting information on demographics, previous and current injury and illness history, footwear and foot posture, knee symptoms, lifestyle, and previous year’s training load; and (3) weekly reports on training load and each participant’s perceptions of cardiorespiratory symptoms, mood, and incidence of illness and injury in the last week. More advanced participation involved connecting participants’ Garmin Connect (Garmin Ltd) data, which included sharing data on running distance, running speed, and heart rate during each training or racing session. Participants added their Garmin Connect information at study recruitment. Garmin data were then automatically uploaded every week when the participant was within the study. Once the participant reaches the end of the study data collection or voluntarily leaves the study, the Garmin data collection link is terminated, ending data upload (Figure 1). Participants could opt out of the study at any time.

#### Ethical Considerations

This study received a favorable ethical review from the University of Nottingham (FMHS 113-1120). All methods were performed following the relevant guidelines and regulations of the Declaration of Helsinki. Before study inclusion, all participants were detailed about the risks and benefits of participation. All participants provided informed consent to participate.

#### Population and Recruitment Strategy

This study aimed to examine recreational runners. The inclusion criteria of this subgroup of the larger “Running Through” [26] study consisted of the following: (1) age ≥18 years; (2) performing running activities; and (3) connecting their Garmin Connect data to the weekly reports. Exclusion criteria consisted of individuals meeting the following conditions: (1) not able or willing to use the internet regularly; (2) diagnosed with an immunocompromised disorder; (3) diagnosed with memory impairment; (4) diagnosed with a neurodegenerative disorder; (5) diagnosed with inflammatory osteoarthritis; and (6) undergone trunk or lower extremity orthopedic surgery in the last 6 months. The larger “Running Through” study consisted of both Garmin and self-report data. Participants were recruited through email, the study website, social media, and word of mouth. Participants resided in the United Kingdom or Europe. All recruitment was performed in English. Participants did not receive compensation for participating in this study. Watch ownership was not known by the research team.

#### Weekly Survey

Participants were sent an encrypted text message or email weekly to report weekly running distance, pace, and incidence of illness and injury [10]. Garmin Connect data also monitored running distance and pace. Garmin monitoring has demonstrated excellent reliability and validity [20].

#### Data Storage

The University of Nottingham’s secure server hosted the research survey tool through the RedCap (Research Electronic Data Capture) service [27]. Data were queried from the secure database using a unique randomized and encrypted identification number.

#### Data Reduction

Watch data were downloaded to an encrypted SQL database using Garmin Connect software (Garmin Ltd). For convenience, these data were combined with the RedCap survey data and tables containing key variables that could be used to link these data. Custom functions were written in R using the DBI and MariaDB packages to interface with the database. The rjson and bit64 packages were additionally used to facilitate the extraction of JavaScript Object Notation format activity data and provide necessary extensions to R’s base data classes. Once data were downloaded, they were aggregated, cleaned, and checked for quality assurance. Data checks were performed through automation and manually.

#### Statistical Analyses

Participant statistics were described using mean (SD) or median (IQR) for continuous variables and frequencies (percentages) for categorical variables. Overall running exposure was calculated in person kilometers.

To assess reliability, intraclass correlational coefficients (ICC_2,1_) were calculated for weekly running distance and running pace between self-reports and weekly reports generated by the Garmin Connect data. Reliability was rated as poor (<0.50), moderate (0.50-0.75), good (0.75-0.90), and excellent (>0.90) [28]. Correlation and Bland-Altman plots were also calculated to assess any systemic measurement error. Analyses were then stratified by sex and age strata (18-40, 41-60, and ≥61 years). All analyses were performed in R 4.1.2 (R Foundation for Statistical Computing) [29], with the psych package for ICC calculations and BlandAltmanLeh for Bland-Altman plots.

## Results

A total of 485 participants linked their Garmin Connect data to the study, with 475 participants included for a total of 3602 participant weeks. Of these, 3 participants were excluded due to lack of follow-up, and another 7 did not run during the collection period (Table 1; the flow chart is available in Multimedia Appendix 1). Participants self-reported running a weekly median of 26.2 (IQR 12.8-39.7) km at a median pace of 6.0 (IQR 5.4-6.7) min/km compared to 25.9 (IQR 4.7-41.8) km running distance at a median pace of 6.1 (IQR 5.2-7.0) min/km (Table 2) recorded by the Garmin watch.

**Table 1:** Participant descriptive statistics.

Variable			Overall (n=475)		Female participants (n=248)		Male participants (n=227)
Age (years), mean (SD)			49.5 (12.2)		51.0 (13.1)		47.8 (10.9)
BMI (kg/m^2^), mean (SD)			24.0 (3.7)		24.2 (3.7)		23.8 (3.8)
Number of weeks followed, median (IQR)			17 (11-23)		15 (10-20)		17 (11-24)
**Smoking, n (%)**							
	Current smoker	10 (2)		6 (2)		4 (1)	
	Ex-smoker	62 (13)		30 (13)		32 (13)	
Cigarettes per day, median (IQR)			9 (3-14)		7 (5-10)		30 (23-37)
Years smoked, median (IQR)			22 (9-35)		14 (2-26)		25 (18-31)
Diabetes, n (%)			6 (1)		1 (1)		5 (3)
Heart disease, n (%)			5 (1)		2 (1)		3 (1)
Cancer, n (%)			14 (3)		10 (4)		4 (2)
Asthma, n (%)			62 (13)		40 (16)		32 (11)
Hay fever (pollen allergies), n (%)			180 (38)		99 (40)		81 (35)
Days of running per week, mean (SD)			2 (1)		2 (1)		2 (1)

**Table 2:** Weekly running descriptive characteristics using Garmin watch data.

Variable	Overall (n=475)	Female participants (n=248)	Male participants (n=227)
Time running (min), median (IQR)	144 (48-238)	139 (43-226)	153 (45-230)
Calories burned (kcal), median (IQR)	1450 (547-2353)	1212 (461-1963)	1755 (671-2840)
Kilometers, median (IQR)	25.9 (4.7-41.8)	22.7 (4.5-40.9)	29.6 (4.9-40.6)
Running pace (min/km), median (IQR)	6.1 (5.2-7.0)	6.7 (5.7-7.7)	5.7 (4.9-6.4)
Average heart rate (bpm^a^), mean (SD)	130 (26)	127 (26)	130 (24)
Maximum heart rate (bpm), mean (SD)	163 (24)	162 (25)	163 (24)

^a^Bpm: beats per minute.

### Reliability

**Figure 2:**
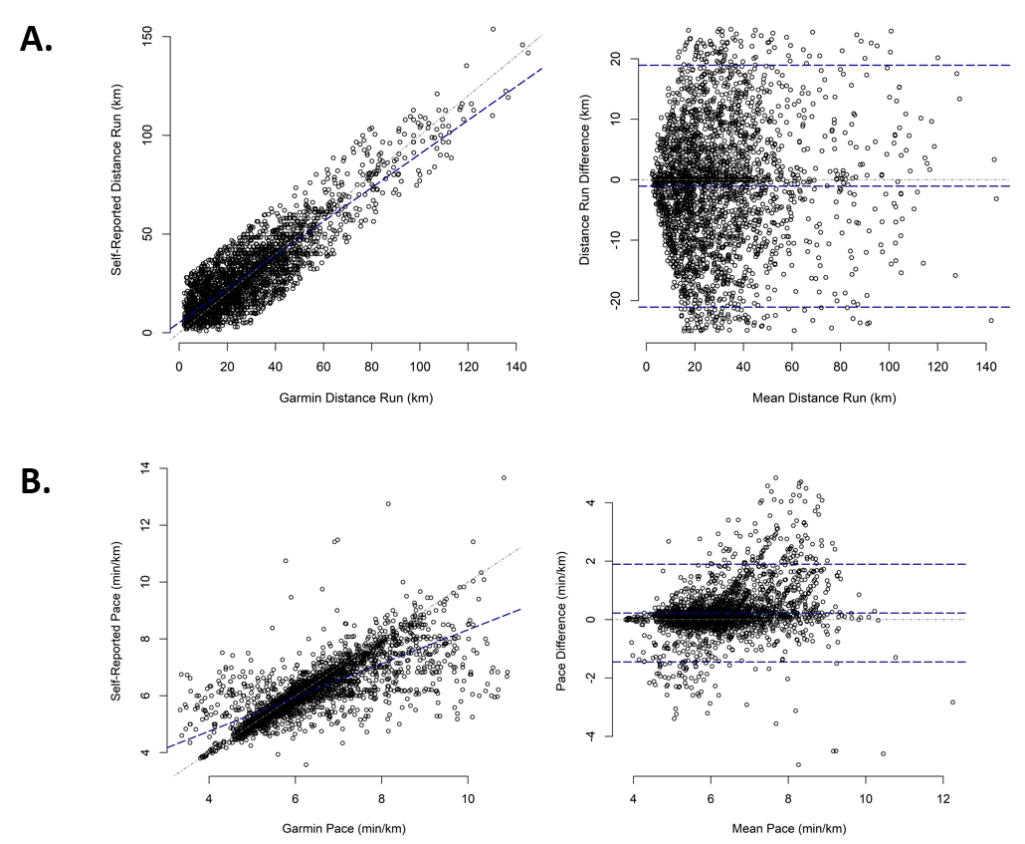
Correlation and Bland-Altman Plots of the Reliability of Self-Report and Garmin Connect Weekly Running Distance and Running Pace. A. Weekly Running Distance (km) B. Weekly Running Pace (min/km).

Weekly distance and pace reliability were rated as good and moderate, respectively, for both sexes and for runners aged 41-60 and ≥61 years. Furthermore, weekly distance reliability was rated as excellent and moderate in runners aged 18-40 years. All results exhibited no discernable systematic bias (Figure 2; Table 3; Multimedia Appendix 1).

**Table 3:** Reliability of weekly self-report and Garmin watch data for running distance and pace.

Group				Self-report		Garmin watch	ICC^a^ (95% CI)
**Weekly distance run (km), median (IQR)**							
Overall (n=475)				26.2 (12.8, 39.7)		25.9 (4.7-41.8)	0.88 (0.87-0.89)
	**Sex**						
		Female (n=248)	24.8 (10.0-39.7)		22.7 (4.5-40.9)		0.86 (0.85-0.87)
		Male (n=227)	29.0 (16.9-41.1)		29.6 (4.9-40.6)		0.89 (0.88-0.90)
	**Age (years)**						
		18-40 (n=113)	27.0 (10.9-43.0)		26.8 (7.2-44.6)		0.93 (0.92-0.94)
		41-60 (n=262)	27.0 (16.0-38.0)		25.9 (11.5-41.2)		0.87 (0.85-0.88)
		≥61 (n=100)	25.0 (13.8-36.2)		24.5 (9.7-39.3)		0.83 (0.80-0.85)
**Average weekly running pace (min/km), mean (SD)**							
Overall (n=475)				6.0 (1.1)		6.1 (2.0)	0.72 (0.69-0.75)
	**Sex**						
		Female (n=248)	6.4 (1.2)		6.7 (1.9)		0.67 (0.62-0.72)
		Male (n=227)	5.7 (0.9)		5.7 (2.1)		0.68 (0.65-0.71)
	**Age (years)**						
		18-40 years (n=113)	5.8 (1.1)		5.7 (2.5)		0.69 (0.65-0.73)
		41-60 years (n=262)	6.0 (1.0)		6.1 (2.5)		0.70 (0.66-0.73)
		≥61 years (n=100)	6.2 (1.2)		6.5 (2.5)		0.74 (0.68-0.78)

^a^ICC: intraclass correlation coefficient.

## Discussion

### Principal Findings

The overall findings of this study indicate that the weekly self-report of running distance by runners wearing a Garmin watch demonstrated good reliability compared to the Garmin watch data. Distance reliability was similar between female and male participants and across age strata, except for participants aged 18-40 years, who demonstrated excellent reliability. Weekly self-report of running pace demonstrated moderate reliability compared to Garmin watch data, with similar reliability observed between sex and age strata. There were no discernable patterns or systematic biases concerning self-reported running distance or pace.

### Comparison to Previous Work

Self-reported running distance exhibited good reliability compared to Garmin data. The reliability is higher in this study compared to a previous study on physical activity (ICC 0.67-0.81) [30]. However, the previous study examined multiple countries and recorded all physical activity beyond running. The homogenous country sample and the focus on running in our study may affect the comparison of these results [31]. Younger adults (aged 18-40 years) demonstrated increased running distance reliability reporting compared to the older age strata (aged 41-60 and ≥61 years). This is comparable with previous research, in which younger adults displayed improved self-report reliability [30]. Younger adults may have a greater aptitude to monitor their running through technology [32]. However, this is only speculative, and further research is required.

Self-reported running pace demonstrated moderate reliability compared to Garmin Connect data. There are currently no studies investigating the reliability of self-reported running pace. However, recreational runners usually train at one pace, with little change at different distances [33]. The moderate reliability observed in this sample may be due to these runners reporting their perceived running pace, with little fluctuation between sessions or weeks. However, specific variances may have occurred in the actual running pace, decreasing the reliability of these data. Previous literature has suggested that instant feedback through the use of heart rate or step cadence can increase a recreational runner’s ability to self-report running pace [33]. However, further work is needed to investigate the efficacy of this approach.

These findings necessitate future research. Participants were recreational runners, and most of them were older than 40 years. Future work is needed to assess the reliability of self-report in comparison to GPS monitoring data in elite runners of all ages and younger populations across different skill or competition levels. All runners in this study already owned a Garmin watch before the study enrollment. Understanding how self-reporting changes among new GPS activity monitor users is needed. Running pace demonstrated moderate reliability in this recreational runner sample. Future research is required to investigate the effectiveness of running pace training on self-report reliability.

### Limitations

As with all studies, there are limitations to this study. First, there is the risk of recall bias due to the weekly intervals for self-reporting, which decreases the precision of these findings. Participants may have not worn or activated their Garmin watch for specific runs, decreasing the reliability of these data. GPS monitoring devices are expensive, causing a high barrier to entry. Such a barrier may add selection bias to this reliability study, decreasing the generalizability of these results to all running populations. Further, the sample in this study comprised recreational runners; therefore, the results are not generalizable to elite runners or populations that solely engage in walking for exercise. Finally, participants used different versions of the Garmin watch. As different watch versions may exhibit different reliability, there is a potential for decreased data precision.

### Practical Applications

Physical activity monitors have effectively enhanced physical activity levels by providing user feedback, facilitating behavior change—following prescribed training—and preventing injuries [34]. The good to excellent reliability of self-reported weekly running distance observed in this cohort of recreational runners across all adult age groups supports previous research indicating that runners can effectively report running loads [30]. These findings strengthen the notion that self-report can be used to reliably monitor runners as they begin or maintain an exercise regimen or return to running following an injury. However, the moderate reliability exhibited for running pace suggests that recreational runners of all ages are not as adept at monitoring their running pace. Incorporating technological monitoring for running pace may be pertinent to maintain prescribed running paces either for specific training regimens (ie, preparing for a race) or when returning to running following an injury.

### Conclusions

Weekly self-report demonstrated good reliability for running distance compared to the Garmin watch data, with similar reliability between sex and age groups. However, the weekly self-report demonstrated only moderate reliability for running pace compared to the Garmin data, with similar reliability between sex and age groups. Sports researchers and scientists can use weekly self-reported running distance in conjunction with Garmin data when quantifying weekly training load. However, caution should be exercised when relying on self-reported running pace to evaluate running intensity in recreational runners. Running pace potentially should be monitored through technological means to increase precision.

### Data Availability

Data and corresponding codes are available within the Open Science Framework [35].

## Appendix

Multimedia Appendix 1 Study flowchart.

## Abbreviations

ACC: intraclass correlational coefficient
RedCAP: Research Electronic Data Capture
