# Supplementary figures and images for: Comparing Self-Reported Running Distance and Pace With a Commercial Fitness Watch Data: Reliability Study
Source: JMIR Form Res. 2024 Jan 4;8:e39211. doi: 10.2196/39211 (PMC10797502; doi:10.2196/39211)

**Appendix 1.** Participant Flow Chart


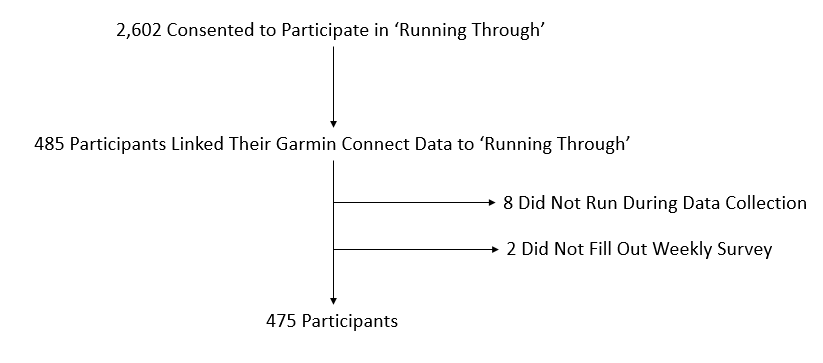

Supplement: Multimedia Appendix 1 [file formative_v8i1e39211_app1.docx]
